# Improved Visual Localization via Graph Filtering

**DOI:** 10.3390/jimaging7020020

**Published:** 2021-01-30

**Authors:** Carlos Lassance, Yasir Latif, Ravi Garg, Vincent Gripon, Ian Reid

**Affiliations:** 1 Electronics Department, IMT Atlantique, 29280 Brest, France; vincent.gripon@imt-atlantique.fr; 2 School of Computer Science, University of Adelaide, Adelaide 5005, Australia; yasir.latif@adelaide.edu.au (Y.L.); ravi.garg@adelaide.edu.au (R.G.); ian.reid@adelaide.edu.au (I.R.)

**Keywords:** graph signal processing, deep learning, visual localization, image retrieval, transfer learning

## Abstract

Vision-based localization is the problem of inferring the pose of the camera given a single image. One commonly used approach relies on image retrieval where the query input is compared against a database of localized support examples and its pose is inferred with the help of the retrieved items. This assumes that images taken from the same places consist of the same landmarks and thus would have similar feature representations. These representations can learn to be robust to different variations in capture conditions like time of the day or weather. In this work, we introduce a framework which aims at enhancing the performance of such retrieval-based localization methods. It consists in taking into account additional information available, such as GPS coordinates or temporal proximity in the acquisition of the images. More precisely, our method consists in constructing a graph based on this additional information that is later used to improve reliability of the retrieval process by filtering the feature representations of support and/or query images. We show that the proposed method is able to significantly improve the localization accuracy on two large scale datasets, as well as the mean average precision in classical image retrieval scenarios.

## 1. Introduction

Can we know where we are in the world based on a single image taken with a cellphone camera? Vision-Based Localization (VBL) [1] answers this problem by retrieving the pose (location and orientation) of a camera based solely on visual information. By going from images to metric localization, VBL enables localization using images alone, which would otherwise be carried out with expensive range based sensor or using complicated image based reconstruction processes.

As with every other scientific discipline, recent advancement in VBL have been lead by deep learning (DL) based methods. Broadly these approaches can be divided into two categories: (a) Pose Regression methods that learn a mapping from images to their poses in a deep network [2,3], and (b) Representation Learning techniques that learn a latent representation of images that is resilient to common nuances such as appearance and viewpoint change [4]. They generally translate physical proximity to closeness in the latent space via contrastive losses. While both of these approaches have their advantages, one of the limiting factors of the regression based techniques comes from its inability to operate in a previously unseen location without needing a costly retraining step. Representation methods, on the other hand, generalize well to new data since the mapping into the learned space is independent of the physical space. Traditional retrieval techniques [5] can be used for visual localization against a set of images with known poses.

In representation learning, Global Positioning System (GPS) has been widely used as a measure of spatial proximity [6]. In the robotics setting, however, there are other cues hinting towards spatial promixity. Images from robots are almost always acquired sequentially using on-board cameras. Similarly, detecting revisits (such as loop closure detection from laser scanners) can establish spatial proximity across time. In this work, we propose a visual representation learning mechanism that takes advantage of these additional spatial proximity cues that are specific to robotic applications. The key insight of this work is that using techniques from Graph Signal Processing (GSP) [7], this additional information can be fused with a “pretrained” deep model without needing any re/fine-tuning. Specifically, we exploit relationships via a graph filter on top of the pre-learned deep representations [4,8]. This work makes the following contributions:

1. We apply the theory and methods of Graph Signal Processing to the problem of visual localization. To the best of our knowledge, this is the first attempt to bring these two areas of research together.
2. Through experiments on real-world datasets, we demonstrate the efficacy of the proposed method in improving localization accuracy with almost no computation overhead at inference.
3. We demonstrate that this method can be applied to traditional image retrieval benchmarks and perform well on them.

The rest of the paper is organized as follows: we present a brief overview of related techniques in Section 2. In Section 3, we formally introduce the proposed method and discuss its properties. In Section 4, we derive and discuss experiments. In Section 5, we conclude the work and discuss future directions.

## 2. Related Work

Visual localization is a well studied problem in the vision community and recent surveys can be found in [1,9]. Traditional methods address the problem using point features using a Bag-of-Words (Bow) approach where each image is represented as histogram of visual word occurrences. Efficient indexing methods then allow retrieving images with similar features and a relate pose computation via the essential matrix. However, such methods can be adversely affected by changes in condition such as weather, time of the day and long term changes such as structure of the scene.

**Deeply learned image representation:** As mentioned in the introduction, various methods in the literature focus on deep learning for generating good embeddings for visual localization, such as NetVLAD [4], GeM [8] and many others. In this work, we build on top of these representations, though the proposed method could be adapted to any latent representation of the images. Its main advantage is that it does not require additional training to perform well. Recent work in robotics [5] has shown that using sequence information in a Bayesian filtering approach, the accuracy of these methods can be vastly improved, even outperforming regression based methods. This technique is also directly applicable to the task of image retrieval. In [10] for example, the authors introduce a new optimization technique that allow them to do a better separation of the support database and to improve the similarity-matching (ranking) phase.

**Graphs in visual localization:** Previous methods  [2,11,12] have made use of graphs to aid visual localization in various ways. One example is the re-ranking of candidates, where a graph performs ranking that takes into account more than one image at a time. This is achieved in [11] by using the closest pair of images and then performing a linear combination of them. In [13] a graph diffusion technique is introduced to improve the ranking phase of image retrieval. Other works such as [2] use techniques like Pose-Graph Optimization (PGO) [14] to take advantage of extra information available (in this case the relative poses of the “test”). Note that these approaches differ from ours as they are used only on the query data. As such, they could be combined with the proposed method, that also considers the support set.

## 3. Proposed Method

In this section, we first provide a very brief overview of graph signal processing then describe the setting in which the current method is applied and then present a formal overview of the GSP techniques used in this work.

### 3.1. Graph Signal Processing

GSP [7] is a mathematical framework that aims at extending harmonic analysis to irregular domains described using similarity graphs. As such, it is possible to define tools such as translations [15], convolutions [16], filtering [17] and wavelets [18] taking into account the complex structure of the inputs. GSP has successfully been applied to domains ranging from neuroimaging [19] to deep learning [16,20,21]. To the best of our knowledge, the present work is the first use of GSP in the context of visual localization.

### 3.2. Problem Setting

We consider the case of autonomous driving where a fleet of vehicles moves around established roads in urban environments. This is a less general case of localizing a freely moving tourist in a city using a mobile phone as the geometry of the road prevents significant view point variations across different traversals. In our case, the change in viewpoints comes from traffic moving in different lanes along the same road. However, there might be significant appearance changes as vehicles can move during any season and any time of the day.

The camera mounted on the vehicle provides a stream of images and we assume that a GPS based location for each image is available for training. The set of images form the database: the collection of images against which matches will be sought based on the learned representation. For image representation, we assume a mapping function that maps each image to a fixed dimensional latent space, with some resilience to viewpoint and appearance changes. For the rest of the section, we use images and latent representation interchangeably to mean a lower dimensional embedding of the original image into a resilient (learned) subspace. A key asset of the latent space is that it linearizes representations. Said otherwise, the linear combinations of latent representations of actual images lead to latent representations of a natural looking (artificial) image.

Once we have the learned representations for each image in the database, new image queries can be processed for localization.

### 3.3. Graph Signals Low-Pass Filtering

We define a graph as G=〈V,A〉, where *V* is the finite set of vertices and A is the weighted adjacency matrix: A[μν] is the weight of the edge between vertices μ and ν, or 0 if no such edge exists. Each vertex represents an image and an edge defines the similarity between two vertices.

In order to avoid irregular artifacts, we consider a normalized adjacency matrix $A=D^{-1/2}WD^{-1/2}$ where W is the direct measure of similarity between two vertices and D is the degree matrix associated with W:

$$D\left[\mu \nu \right]=\left\{\begin{array}{cc} \sum_{k\in V}W[\mu k] & \mathrm{if}\;\mu =\nu \\ 0 & \mathrm{otherwise} \end{array}\right.\;.$$

Note that this normalization is only well-defined if the graph has no isolated vertex, what we consider to be true in the following. Given a graph G=〈V,A〉, consider a matrix $s\in R^{V\times d}$, where d∈N. We refer to s as a signal in the remaining of this work, and typically we consider s to be composed of the concatenation of latent representations of images corresponding to vertices in *V* such that a row of s corresponds to an image in the database. We define the graph low-pass filter [22] $h_{G}\left(s\right)$ of s as:

(1) $$h_{G}\left(s\right)={\left(I-aL\right)}^{m}s,$$

where *m* is the number of times the filtering is applied, I is the identity matrix and L=I−A is the Laplacian of the normalized matrix A. This creates a low-pass filter that allows us to smooth the support database and obtain better results when querying it. The parameter *a* controls the intensity of each filtering operation, and we use a=0.1 in this work.

Let us explain briefly why this can be called a low-pass filter in s. First note that because L is symmetric and real-valued, it admits |V| eigenvalues (where |·| denotes the cardinal). The way L has been normalized, all these eigenvalues are between 0 and 2. Other interesting properties include that the eigenspace associated with the eigenvalue 0 is composed of constant vectors and 2 is not an eigenvalue if the graph is not bipartite.

So, considering the graph is not bipartite, multiplying the signal by I−aL or one of its positive powers has the effect of diminishing the influence of all components of the signal that are not aligned with a constant vector, while maintaining the latter. As a result, the difference between representations of neighboring vertices in the graph is reduced. This operation has the effect of smoothing the signal, in the sense that the *i*-th column of the smoothed signal is such that the difference in values between two (strongly) connected vertices is going to be smaller than that before smoothing. In the extreme case of smoothing multiple times (i.e., large *m*), this would eventually have the effect of averaging all representations in connected components of the graph. Let us emphasize that the output vectors of the proposed graph filtering method are linear combinations of the input feature vectors. The main interest of using the framework of GSP is that it allows to offer a nice interpretation of the chosen linear combination. Also, we consider our work to be a first step towards many possible uses of the rich literature on GSP to improve performance of VBL.

**Figure 1:**
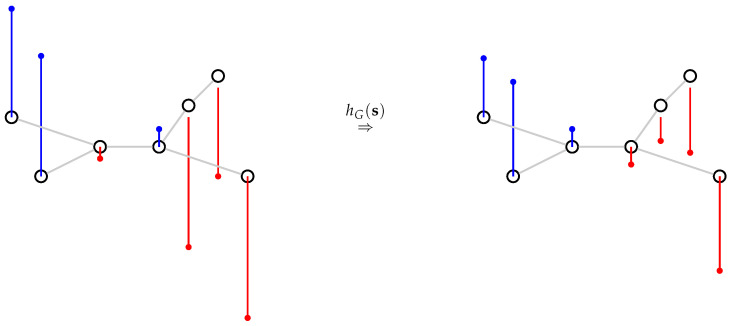
Illustrative example of the graph filter. The signal is represented by the blue(positive) and red(negative) bars.

In brief, the low-pass graph filter we implement has the effect of smoothing the signal values, taking into account strongly connected vertices in the graph. As a result, outliers are smoothed using similar images in the graph. In this work, we consider the vertices to be either the database items or the query set items. In both cases, the goal is to use graph filtering to reduce the noise in the latent representations by incorporating additional information. This is illustrated in Figure 1, where we consider a unidimensional signal represented using blue (for positive values) and red (for negative values) bars. Before filtering (on the left), neighboring vertices can have large variations in their signal values. After filtering (on the right), these variations are lowered. Note that the parameter *m* in Equation (1) controls the intensiveness of smoothing: when *m* is small (i.e., almost 0), ${\left(I-aL\right)}^{m}$ becomes close to the identity matrix and the filtering has almost no effect. When *m* is large (i.e., m≫1), ${\left(I-aL\right)}^{m}$ becomes an averaging matrix on each of its connected components.

### 3.4. Graph Definition

In order to improve the accuracy of VBL, we need to make sure that the edges of the graph are well chosen to reflect the similarity between two images represented as vertices. As our main goal is to exploit extra information available at the database, in this work we consider three different sources:

- Metric distance (dist): the distance measured by the GPS coordinates between vertices μ and ν;
- Sequence (seq): the distance in time acquisition between two images (acquired as frames in videos);
- Latent similarity (latent_sim): the cosine similarity between latent representations.

The matrix W can therefore be derived from the three sources as:

$$W=W_{\mathrm{dist}}+W_{\mathrm{seq}}+W_{\mathrm{latent}\_\mathrm{sim}}.$$

#### 3.4.1. Metric Distance

In order to transform the metric distance into a similarity, we use an exponential kernel. This is parametrized by a scalar α that controls the sharpness of the exponential and a threshold parameter $max_{distance}$ that cuts edges between distant vertices:

$$W_{\mathrm{dist}}\left[\mu \nu \right]=\left\{\begin{array}{cc} e^{\alpha dist_{\mu ,\nu }} & \mathrm{if}\;dist_{\mu ,\nu }<max_{distance}\\ 0 & \mathrm{otherwise} \end{array}\right..$$

Note that the choice of an exponential kernel may seem arbitrary, but is often used in the area of Graph Signal Processing [7].

#### 3.4.2. Sequence

To exploit the information of time acquisition of frames, we use the function seq(k,μ,ν) which returns 1 if the frame distance between μ and ν is exactly *k* and 0 otherwise. We then build a matrix $W_{\mathrm{seq}}$ parametrized by scalars $\beta_{k}$ and $k_{max}$:

$$W_{\mathrm{seq}}\left[\mu \nu \right]=\sum_{k=1}^{k_{max}}\beta_{k}seq\left(k,\mu ,\nu \right).$$

#### 3.4.3. Latent Similarity

Finally, we define a matrix $W_{\mathrm{latent}\_\mathrm{sim}}$ for the latent representations cosine similarity. This is parametrized by a scalar γ that controls the importance of the latent similarity. We only compute this similarity if either the distance similarity or the sequence similarity is nonzero:

$$W_{\mathrm{latent}\_\mathrm{sim}}\left[\mu \nu \right]=\left\{\begin{array}{cc} \gamma sim(\mu ,\nu ) & \mathrm{if}\;W_{dist}\left[\mu \nu \right]>0\\ & \mathrm{or}\;W_{seq}\left[\mu \nu \right]>0\\ 0 & \mathrm{otherwise} \end{array}\right.,$$

where sim is the latent similarity function. In this work we use the cosine similarity ($sim_{\cos}$), but any similarity function could be used.

## 4. Results

### 4.1. Visual Localization

We first present some results for the visual localization task using publicly available data. First we describe how the dataset was collected in the following section, then we describe how we choose our hyperparameters and finally we display our results.

#### 4.1.1. Dataset Generation

**Figure 2:**
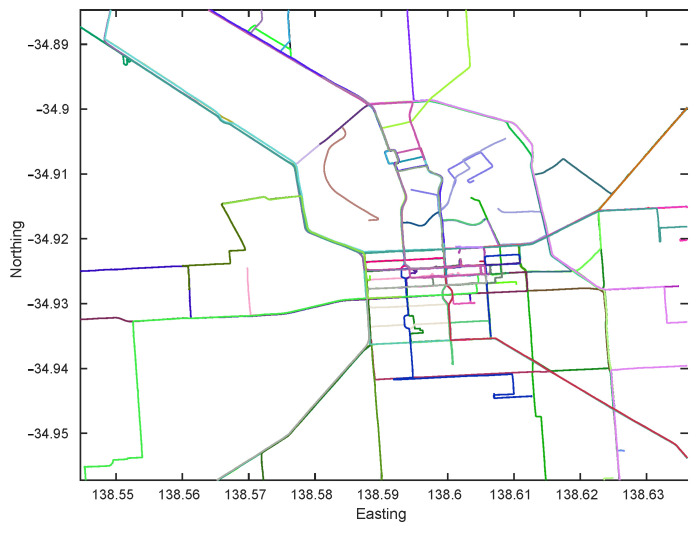
GPS Tracks of the image sequences collected around Adelaide CBD from Mapillary.

**Figure 3:**
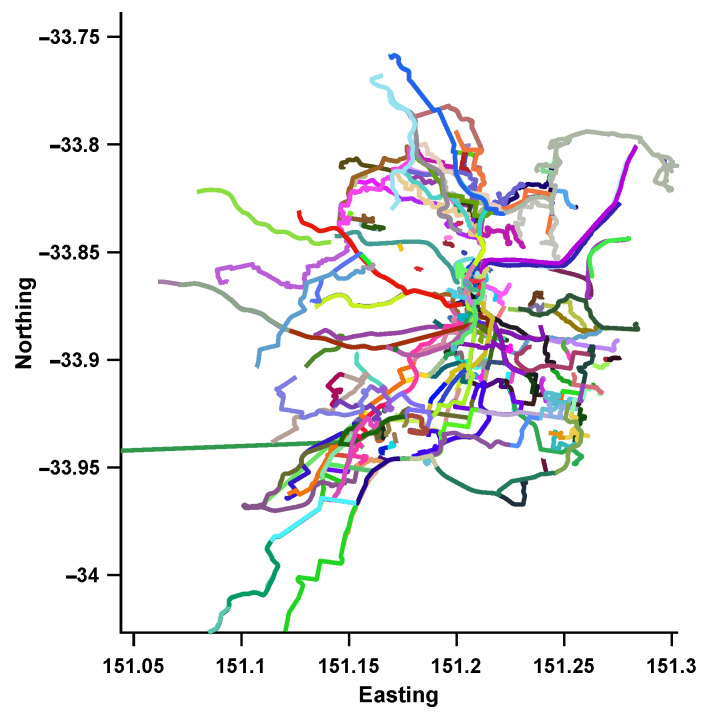
GPS Tracks of the image sequences collected around Sydney from Mapillary.

To verify the effectiveness of our method in an autonomous driving context, the first step is to use an appropriate dataset that is collected from roads and is large enough to demonstrate appearance changes and (limited) viewpoint changes due to road structure. We collect images from the Mapillary API (https://www.mapillary.com/developer/api-documentation/), which contains crowd sourced data over years for major roads. To show the generalization ability of the proposed work, we collect road imagery from two Australian cities. The first covers the Central Business District (CBD) area of Adelaide, Australia and spans an area of roughly 10 km${}^{2}$. Since the data is publicly sourced, there is a lot of viewpoint, illumination and dynamic changes (cars, pedestrian, etc). The second set is collected around the Greater Sydney region and covers an area of around 200 km${}^{2}$. We note that the data collected for the Greater Sydney region contains some sequences that were generated using different equipment (panoramic cameras) or different positioning (camera pointed to a vehicle window instead of the windshield) from the ones used during the training of the NetVLAD network, which combined with the total area covered by the database creates a much more challenging problem. In addition to imagery, the collected data provides sequence information and GPS. The GPS tracks for the collected data are shown in Figure 2 and Figure 3.

In the rest of the experiment section, we use the terminology of visual localization, that is, support database refers to the reference database, validation and test queries refer to query inputs.

To split the Adelaide dataset in support/validation/test we randomly choose 4 sequences for validation and 5 sequences for testing. For the Sydney database, we choose 5 sequences that could be retrieved with reasonable performance using our pre-trained NetVLAD (named easy query) and 5 sequences at random (hard query). Using GPS as ground truth, we remove all examples from the query sets that are further than 25 m from the support dataset (i.e., there are no examples in the support set in a 25 m radius from them). The statistics for each dataset are summarized in Table 1.

#### 4.1.2. Parameter Definition

For all the results in the subsequent sections we use the same parameters, which were obtained using a grid search and keeping the best score on the Adelaide validation query. We use the Adelaide test query to ensure that the parameters are not overfitted to the validation query. Also note that we use the same parameters for both cities to further validate the fact that we do not need additional training/parameter search for each new city. The parameters are $\alpha =0.1,\;\beta_{1}=0.75,\;\beta_{2}=0.0625,\;\beta_{3}=0.015,\;k_{max}=3,\;\gamma =0.66,\;m=19$.

#### 4.1.3. Application to VBL

The method is applied to VBL using the following steps

1. Features are extracted using [4];
2. Graphs are generated for support, query or both using the previously described graph inference method;
3. If graphs exist for a set, the features of the set are then filtered using the previously described methodology;
4. Localization of a query image is then defined by the nearest example in the support database (either using features from step 1 or 3, depending on where graph filtering is applied).

#### 4.1.4. Results

We test the graph filter in three different cases. First the extra data is available only for the support, second it is available only for the query and finally it is available in both cases. In each case we report two metrics, the median localization error over all the queries and the percentage of localizations that have less than 25 m error.

First we perform the tests on the Adelaide dataset and present the results in Table 2. The graph filter was able to increase performance, even when applied only on the query database, and as expected, adding the graph filter during both query and support gave the best results. Recall that the parameters were defined based on the validation query, under the case where the extra data is available only for the support database.

Second we validate that the operation can be used on another city and that we do not need to perform an additional grid search for the new data. The results are presented in Table 3. As expected the graph filter allowed us to get better performance in both median distance and accuracy, while using the parameters optimized for the Adelaide dataset. This is inline with our goal to obtain a processing methodology that does not require to be retrained or re-validated for a new city. We note that the performance of the hard query set is not inline with a good retrieval system (several kilometers from the correct point), but it is included to show that our method allows us to increase the performance both when the NetVLAD features are already very good for the task and when they are very bad.

#### 4.1.5. Ablation Studies

To verify that each part of the graph is important, we perform ablation studies using the Adelaide test query. The results are presented in Table 4. The table shows that different sources of information are important, with each one adding to increase in performance. Metric distance and sequence are the most important features and latent similarity is more of a complementary feature (this is expected, as it is being thresholded by the other two features). This is encouraging since in the absence of any other external information (GPS, etc), one can rely on the sequential nature of data collection to get a boost in localization performance. This information is readily available in a robotics setting.

**Figure 4:**
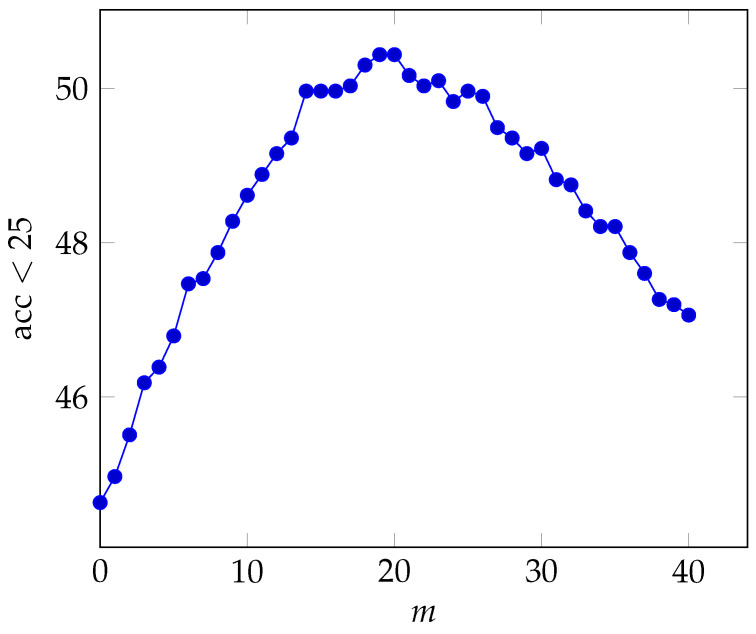
Effect of the parameter *m* on the retrieval accuracy under 25 m for the Adelaide test query.

In the next experiment, we demonstrate the effect of successive filtering. This is achieved by varying the value of m, the number of times the graph filtering is applied. Theoretically, this should help increase the performance until it hits a ceiling and then it should start to slowly decrease (as it enforces connected examples of the database to be too similar to each other). The results are presented in Figure 4. As can be seen, there is a clear pattern of increased performance until m=20 after which the performance starts to degrade. It should be noted that even for m=40 the graph filter still performs better than the baseline (m=0).

### 4.2. Image Retrieval

As a visual localization problem can be seen as an application of Image Retrieval, we test our method in classical Image Retrieval scenarios to verify its genericity. We use the revisited Oxford and revisited Paris datasets [23] with the features from [8]. In this case we do not have the physical distance between the images to properly create $W_{\mathrm{dist}}$ or the image sequence to generate $W_{\mathrm{seq}}$. We therefore use the objects names as classes and our $W_{\mathrm{dist}}$ is composed of only 1 if μ,ν are from the same object and 0 otherwise. Note that in this way, we differ from traditional methods as they tend to not consider this additional information during training or testing and therefore comparison with other methods is not entirely fair. All the other parameters are the same as in the localization scenario.

In the scenario of Image Retrieval our approach can be categorized as diffusion-based. In the literature there are diffusion methods that are used during the ranking phase with *k*-nn graphs [13] or that add an additional GCN [24] component that has to be trained in an unsupervised way [10]. The former only affects the ranking of supported images for a query (i.e., the way we evaluate the similarity between query and support set, by applying graph diffusion) while the latter acts in both ranking (which becomes GCN-diffusion based) and feature definition (i.e., it learns a GCN-diffusion in order to modify the original features from [8]). In summary, our main contribution is the graph construction (taking advantage of the class data that is available on the support set) and our smoothing/diffusion technique that is based on a low-pass filter.

The results are presented in Table 5. The baseline for our comparison are the results obtained using cosine similarity to rank the features obtained from [8]. We then compare using our filter to generate new features, with the ranking diffusion method from [13] (lines 2 and 3). Finally we also compare the combination of the features generated from our filter with the ranking diffusion from [13] to the method described in [10] which uses the same base features and learns a new feature set and ranking function. In summary, our method was able to increase the mean average precision, with similar results to the approach from [13]. When using in combination with [13] we achieve a similar performance on the Paris dataset to a state of the art approach [10] that requires training an additional GCN network.

## 5. Conclusions

This work showed that using techniques from Graph Signal Processing, the performance of visual based localization and image retrieval can be improved by incorporating additional available information. This additional information acts on the latent representation by making it smoother on a graph designed using all available information, leading to a boost in localization. One of the encouraging observations of the work is that this additional information can take the form of a simple temporal relationship between surrounding images acquired in a sequence, and still lead to a significant increase in performance.

In future work, we would like to use the graph during the localization inference, to add temporal consistency to the position inference and also to train the filter operation in an end-to-end fashion.

## Figures and Tables

**Table 1:** Summary of the vision-based localization datasets used in this work.

City	Adelaide	
	**# Sequences**	**# Images**
Support Database	44	24,263
Validation Query	4	2141
Test Query	5	1481
	**Sydney**	
	**# Sequences**	**# Images**
Support Database	284	117,860
Easy Query	5	1915
Hard Query	5	2285

**Table 2:** Results under different graph filter conditions for the Mapiliary Adelaide dataset. GF means Graph Filtering. The best performance for each row is bolded.

Measure	None	GF Database	GF Query	GF D + Q
Validation				
acc < 25 m	66.84%	76.09%	69.92%	**79.22%**
median distance	8.76 m	**6.90 m**	13.04 m	8.86 m
Test				
acc < 25 m	44.63%	50.44%	46.32%	**52.06%**
median distance	110.66 m	24.30 m	41.84 m	**22.66 m**

**Table 3:** Results under different graph filter conditions for the Mapiliary Sydney dataset. GF means Graph Filtering. The best performance for each row is bolded.

Measure	None	GF Database	GF Query	GF D + Q
Easy				
acc < 25 m	49.45%	55.28%	55.46%	**63.75%**
median distance	28.25 m	14.12 m	18.77 m	**11.93 m**
Hard				
acc < 25 m	13.87%	17.33%	16.54%	**24.86%**
median distance	4000 m	3253 m	3180 m	**1700 m**

**Table 4:** Ablation study on the Mapiliary Adelaide test query. The best result per column is bolded.

$W_{\mathrm{dist}}$	$W_{\mathrm{seq}}$	$W_{\mathrm{latent}\_\mathrm{sim}}$	Median Distance	acc < 25 m
			110.66 m	44.63%
X			29.26 m	49.42%
	X		39.11 m	47.47%
X		X	28.41 m	49.56%
X	X		24.35 m	50.17%
	X	X	37.34 m	47.74%
X	X	X	**24.30 m**	**50.44%**

**Table 5:** mAP retrieval results comparison, results that do not include our filter are extracted as is from [10]. Best results per column are bolded.

		rOxford		rParis	
**Features**	**Ranking**	**Medium**	**Hard**	**Medium**	**Hard**
[8]	$sim_{\cos}$	64.7	38.5	77.2	56.3
[8]	[13]	69.8	40.5	88.9	78.5
[8] + Our filter	$sim_{\cos}$	70.58	47.67	87.77	76.04
[8] + Our filter	[13]	71.41	51.27	91.54	81.85
[8] + [10]	[10]	**77.8**	**57.5**	**92.4**	**83.5**

## Data Availability

Restrictions apply to the availability of the data forthe Sidney and Adelaide datasets as the data was obtained from Mapilliary via their API https://www.mapillary.com/developer/api-documentation/. Revisited oxford and revisited paris datasets are available at https://github.com/filipradenovic/revisitop.
